# ‘If he thought that I was going to go and hurt myself, he had another thing coming’: Treatment experiences of those with large to massive rotator cuff tears and the perspectives of healthcare practitioners

**DOI:** 10.1177/02692155241235338

**Published:** 2024-02-28

**Authors:** Kathryn Fahy, Rose Galvin, Jeremy Lewis, Karen McCreesh

**Affiliations:** 1 School of Allied Health, 8808 University of Limerick, Limerick, Munster, Ireland; 2 Department of Clinical Therapies, 150229 University of Limerick Faculty of Education and Health Sciences, Limerick, Munster, Ireland; 3 Therapy Department, Central London Community Healthcare National Health Service Trust, Central London Community Healthcare National Health Service Trust, London, UK; 4 School of Allied Health, 150229 University of Limerick Faculty of Education and Health Sciences, Limerick, Munster, Ireland

**Keywords:** Large to massive rotator cuff tears, qualitative study, treatment experiences, healthcare professionals, shoulder pain

## Abstract

**Objective:**

To explore the treatment experiences of those diagnosed with large to massive rotator cuff tears and the perspectives of healthcare practitioners providing their care.

**Design:**

A qualitative descriptive study using reflexive thematic analysis.

**Setting:**

In-person focus groups were undertaken in a clinical setting (private practice [*n* = 1]; public outpatient [*n* = 2]). Semi-structured interviews were conducted online via Microsoft Teams.

**Participants:**

Patients diagnosed with these tears (*n* = 12) and healthcare practitioners (*n* = 11).

**Results:**

Two interlinking themes were identified based on the care received and provided for patients with symptomatic large to massive rotator cuff tears:

1) *Positive treatment experiences and management:* Education, clear communication and reassurance around prognosis were the foundation of positive patient–clinician care. Sub-themes of pain relief, exercise prescription and confidence in their pathway underpinned this experience. This proficiency in care was affirmed by some *healthcare practitioners* who spoke about the importance of confidence and experience in their management plan even in times of poor progress.

2) *Negative treatment experiences and management:* Uncertainty, delays and exacerbation of pain flawed the patient–clinician care. Sub-themes of inappropriate pain relief, inappropriate exercise prescription and uncertainty impacted their care. Some *healthcare practitioners* acknowledged knowledge gaps led to uncertainty especially when choosing the next step of care and were quick to escalate care to deflect this uncertainty.

**Conclusions:**

The findings suggest discordance exists between the patient's experiences and expectations when the delivery of care was by less experienced and confident healthcare practitioners in the management of this condition. This highlights the need for improved education and support for healthcare practitioners.

## Introduction

Symptoms attributed to tears in the rotator cuff are considered to be one of the most prevalent conditions affecting the adult shoulder. As the population ages, these tears commonly increase in size, becoming large to massive and believed to become symptomatic in two of every three people over the age of 80 years.^1,2^ Although the exact relationship between structural changes and symptoms remains uncertain, ageing, lifestyle, genetics, co-morbidities, ergonomic factors and certain work-related activities are implicated in rotator cuff tendon degeneration, tears and concomitant glenohumeral osteoarthritis.^3^ Symptomatic, large to massive rotator cuff tears are associated with persistent day and night pain, weakness detrimentally impacting independence, participation and quality of life.^4,5^

There is still uncertainty regarding the best treatment for these tears. This is due to the limited, variable and inconclusive evidence concerning the relative effectiveness of surgical versus non-surgical treatment approaches^6^ with no gold standard emerging.^7^ The success of surgical treatment is often determined by the patient's age, size of the tear and reparability of the tendon, short- and long-term functional demands of the patient, expectations and concomitant co-morbidities.^8^ Non-surgical interventions include combinations of exercise and education with or without pain management.^9^ There is also uncertainty regarding the parameters of non-surgical treatment and the level of supervision required, with duration cited between three to twelve months.^7^ A recent systematic review and meta-analysis highlighted a paucity of high-quality research on the role of exercise in the management of large to massive rotator cuff tears.^10^ Fahy et al. identified substantial discrepancies in the content and reporting of the exercise intervention in the published research adding another layer of uncertainty when comparing interventions.

Notwithstanding the uncertainty regarding the optimal management of large to massive rotator cuff tears, even less is understood regarding patients’ experiences, beliefs and expectations. This is further compounded by a poor understanding of the perspectives of healthcare professionals in treating this cohort. The value of qualitative research to improve understanding of patients’ experiences and of the complex processes involved in treatment outcomes is well recognised and accepted.^11^ A recent qualitative synthesis exploring the experiences of people living with shoulder pain^12^ highlighted a significant gap in knowledge with no study to date investigating the treatment experience of individuals with large to massive rotator cuff tears and the influence it may have on their chosen intervention and perception of outcomes. People with large to massive rotator cuff tears tend to be significantly older, and more likely to have co-morbidities, than the general cohort of people with rotator cuff disorders.^1^ Frequently, as mentioned this manifests as a significant loss of shoulder function, loss of independence and reduced quality of life.^4,5^ It is important to determine whether there are specific differences experienced by those with large to massive rotator cuff tears compared to shoulder pain, and what influence the views and experiences of the clinicians who care for them have on their treatment experience and outcome.

As such, an exploration of the care experiences of patients with large to massive rotator cuff tears and healthcare practitioners managing their care might assist in the provision of care that is acceptable, reproducible, relevant and achievable for both patients and healthcare practitioners. To date, there are no qualitative studies that focus on the experiences and current practices of healthcare practitioners in managing this cohort and the experience of patients during their treatment.

Thus, this study aims to explore the treatment experiences of those diagnosed with symptomatic large to massive rotator cuff tears and the perspectives of healthcare practitioners providing this care.

## Methods

### Study design

A qualitative descriptive study design using reflexive thematic analysis was employed for this study.^13^ This approach was chosen as it aligns with naturalistic inquiry and complements our study aims of uncovering the experiences and views of people with large to massive rotator cuff tears and healthcare practitioners’ clinical management.^13^ It aims to offer insights into how a given context makes sense of a given phenomenon. The sample size for this study adhered to guidance from Malterud et al. since ‘information power’ appears more suitable than ‘data saturation’ in the context of reflexive thematic analysis.^14^ Guided by the aim of the study, the specificity of the sample of participants, the established theory, the quality of the data and the analysis strategy, we sought to recruit 14 patients with large to massive rotator cuff tears in this study to allow for dropouts and eleven healthcare practitioners. To ensure rigorous conduct and reporting, this study was reported in accordance with Consolidated Criteria for Reporting Qualitative Research^15^ (Supplementary File 1).

### Recruitment and participants

A mixed purposive sampling technique was used in this study.^16^ Criterion purposive sampling was used to recruit patients with large to massive rotator cuff tears based on predefined inclusion/exclusion criteria.^16^ For these patients, recruitment letters and study information sheets with contact details for the study investigators were sent to the participating healthcare practitioners to display in their clinics. In addition, open recruitment through social media accounts of the study authors was employed (i.e., Twitter, Instagram).

Patients with large to massive rotator cuff tears were included if they met the following inclusion criteria: over 55 years of age with a patho-anatomical diagnosis confirmed by magnetic resonance imaging of a symptomatic large to massive rotator cuff tears (>5 cm, 2 or more tendons involved), having undergone or awaiting surgical intervention or conservative management. Individuals were excluded if they had: an acute tear with no history of shoulder pain, a shoulder fracture, adhesive capsulitis or any neurological signs or if they were unwilling or unable to give informed consent. Demographic characteristics of the participants with large to massive rotator cuff tears are outlined in Table 1*.*

**Table 1.** Demographic characteristics of people with large to massive rotator cuff tears.

Participant	Age range	Gender	Duration of symptoms	Affected shoulder	Dominant upper limb	Intervention
1-FG1-M1	66–75	Male	6–12 m	Right	Left	SCR
2-FG1-M2	76–85	Male	6–12 m	Right	Right	SCR
3-FG1-M3	66–75	Male	6–12 m	Left	Left	RC Repair
4-FG1-F4	76–85	Female	6–12 m	Right	Right	RSA
5-FG1-F5	66–75	Female	2 years	Right	Right	RSA
6-FG1-M6	66–75	Male	6–12 m	Right	Right	SCR
7-FG2-F2	76–85	Female	6–12 m	Right	Right	Conservative
8-FG2-F3	66–75	Female	6–12 m	Right	Left	RC Repair
9-FG2-M4	55–65	Male	6–12 m	Left	Right	Awaiting RC Repair
10-FG3-F1	66–75	Female	2 years	Right	Right	RC Repair
11-FG3-M2	55–65	Male	6–12 m	Left	Left	Conservative
12-FG3-M3	55–65	Male	6–12 m	Left	Right	Conservative

FG: focus group; SCR: superior capsular release; RC: rotator cuff; RSA: reverse shoulder arthroplasty.

Healthcare practitioners were recruited using snowball purposive sampling.^16^ It is a non-random sampling technique also referred to as referral sampling where existing research participants recruit another participant from their acquaintances who is information-rich for the specific purpose of the study.^17^ Snowball sampling also facilitates the continuation of sampling through social networking.^18^ A stratified sample of healthcare practitioners was sought to ensure a range of clinicians (surgeons, general practitioners, physiotherapists) both private and public with a range of experience. A recruitment email and study information sheet were sent to gatekeepers at the Irish Shoulder and Elbow Society, the Irish College of General Practitioners and the Irish Society of Chartered Physiotherapists for distribution among their members. An email to facilitate recruitment was also distributed through a local general practitioner network. A social media post for healthcare practitioner recruitment was also distributed and shared among the research team's professional networks. Healthcare practitioners were eligible to participate if they were healthcare practitioners (orthopaedic surgeons, physiotherapists, general practitioners) dealing with people diagnosed with large to massive rotator cuff tears with a minimum of five years of experience in shoulder care and had managed at least three patients with large to massive rotator cuff tears in the previous year. In Ireland, these are the core healthcare practitioners involved in the management of individuals with large to massive rotator cuff tears. Demographic characteristics of the HCPs are presented in Table 2.

**Table 2.** Demographic characteristics of HCPs.

Participant	Age **r**ange	Gender	Occupation	Years of **e**xperience	% Caseload shoulder specific
HCP1	30–39	Female	Physiotherapist (private)	6–10 7years	76–100%
HCP2	50–54	Male	General Practitioner (GP practice)	15+ years	0–25%
HCP3	40–49	Female	Orthopaedic surgeon (private)	11–15 years	76–100%
HCP4	30–39	Female	Physiotherapist (public)	11–15 years	51–75%
HCP5	40–49	Female	Physiotherapist (private)	11–15 years	0–25%
HCP6	30–39	Male	Physiotherapist (public)	6–10 years	0–25%
HCP7	66–75	Male	General Practitioner (GP practice)	15+ years	0–25%
HCP8	40–49	Male	Orthopaedic surgeon (public)	11–15 years	51–75%
HCP9	30–39	Male	General Practitioner (GP practice)	0–15 years	0–25%
HCP10	40–49	Male	Physiotherapist (private)	15+ years	0–25%
HCP11	40–49	Male	Orthopaedic surgeon (public)	15+ years	76–100%

### Ethical considerations

Ethical approval was granted by the University of Limerick ethics committee *(2022_03_11_EHS).* All potential participants received an information sheet that outlined the purpose of the study, study procedure, study risks and benefits and confidentiality and anonymity around the reporting and dissemination of the study information. Completion of the written informed consent form was a prerequisite for study participants. All participants were made aware that they had the right to withdraw at any point and their participation was voluntary.

### Data collection and interviews

In-person focus groups were held with patients with large to massive rotator cuff tears and were audio recorded. Focus groups allow group members to build on each other's input, facilitating greater discussion and debate among the participants.^19^ Focus groups were undertaken in a clinical setting (private practice clinic [*n* = 1]; public outpatient hospital setting [*n* = 2]). For healthcare practitioners, semi-structured individual interviews were conducted online via Microsoft Teams. The choice of online video-recorded interviews provided more flexibility for scheduling for busy healthcare practitioners and enhanced the geographical spread of healthcare practitioners included.

An interview guide with open-ended questions was used to structure the focus groups and semi-structured interviews. Open-ended questions allowed patients with large to massive rotator cuff tears to explore and discuss their treatment to date and allowed healthcare practitioners to describe their experiences to date in the clinical management of this presentation. The interview questions were developed by the researchers by reviewing existing literature related to patients’ and HCPs’ experiences in managing other shoulder pathologies.^12,20^ The interview guide used for patients with large to massive rotator cuff tears focused on their treatment experience with a specific focus on pain, exercise and their interactions with their treating practitioner. Topics covered in the interviews with healthcare practitioners included their experience in managing this cohort and how the care pathway could be improved. The interview was pilot-tested with one participant with a large to massive rotator cuff tear and one physiotherapist to assess the clarity and appropriateness of questions, and the flow of the interview. The interview guides are provided as supplementary material in this manuscript.

All interviews were conducted by KF, a female physiotherapist trained in Ireland to masters’ level, who also works in sports and private practice in Ireland. This study forms part of KF's doctoral research. KF had no prior established relationship with any of the participants in this study. Participants were informed that KF was a clinician who was conducting research relating to large to massive rotator cuff tears. Her reason for conducting this research was underpinned by her clinical interest in this condition.

### Data analysis

At the end of each interview, the audio recording was fully transcribed verbatim by KF. NVivo software package (version 12) was used to import transcripts, organise, store and retrieve data for analysis. Reflexive thematic analysis, an inductive approach to data analysis, was used in this study. An inductive approach was chosen because it is data-driven rather than trying to fit into an existing code frame or the researchers’ analytic presumptions.^21^

The thematic analysis was conducted using a realist/essentialist paradigm which focuses on reporting experiences and meaning to the individual.^21^ Methodological rigour was addressed using the following strategies: adhering to the ‘Twenty questions to guide assessment of thematic analysis research quality’,^13^ extensive field notes cross-checked with the data set, documentation of the personal reflections of the researcher relating to her experience as a physiotherapist, her beliefs and values to ensure reflexivity through transparency and trustworthiness.^22^ Throughout the analysis process, detailed discussions between (KF) a novice qualitative researcher and (KMcC and JL) both experienced qualitative researchers to enhance understanding and clarity in the interpretation of the data. The lead researcher (KF) maintained a reflexive diary which outlined the impact of her status as a physiotherapist with a special interest in shoulder presentations on the interviews and data analysis.

Thematic analysis is divided into six clear systematic phases^13,21,23^ which were conducted by (KF) with critical review by KMcC, JL and feedback from RG as follows in Table 3*.* Finally, a narrative reflective report was provided by KF and reviewed by KMcC to ensure a clear audit trial of methodology during the research phase.^21^

**Table 3.** Six-phase process of thematic analysis.

Phase 1 – Data familiarisation	Full and prolonged engagement with the data was achieved through conducting and transcription of the interviews from both the MS audio files and a backup voice recorder for increased clarity. Re-reading the interview transcripts alongside the audio files was then conducted.
Phase 2 – Generating initial codes	The lead author (KF) coded all the transcripts beginning with the HCP semi-structured interviews, followed by the focus group interviews. These were coded through an open coding approach and sections of the transcripts were also coded by KMcC at this stage to refine and agree the code. This resulted in a list of codes that represented both patterning and diversity of relevant meaning in the data.
Phase 3 – Generating Themes	KF, KMcC and JL reviewed and collated all potentially relevant codes to generate candidate themes which were channelled by the research question and study aims and aided by drawing thematic maps.
Phase 4 – Reviewing potential themes	This was accomplished by reviewing the link between the candidate themes against the original codes, the original dataset and against one another to ensure themes were distinct yet related to one another.
Phase 5 – Defining and naming theme	KF, KMcC and JL refined the themes through the discussion of theme definitions to clarify the scope of the themes and editing the theme titles to ensure they were descriptive of the central organising concept of the theme.
Phase 6 – Producing the report	Included the write-up of the narrative report by KF and then deciding how quoted data extracts would be best presented with feedback from RG. Pseudonyms were used to ensure de-identification of all participants while reporting the data.

Final reports and findings were provided to participants for feedback and data verification. No data that could identify study participants were used within the analysis or presentation of the study.

## Results

In total, 14 patients with large to massive rotator cuff tears consented to take part, with one (aged 49 years) under the age range to meet the inclusion criteria. Another potential patient underwent knee replacement surgery so was unable to attend the focus group. Therefore 12 patients with large to massive rotator cuff tears were interviewed using a focus group approach. Eleven healthcare practitioners, all working in the Republic of Ireland were included and undertook semi-structured interviews. Demographics of all participants can be seen in Table 1 and Table 2. The average duration for the large to massive rotator cuff tears focus groups was 60 min (range: 56–63). The average duration of the healthcare practitioners interviews was 39 min (range: 27–46). Field notes were taken during all the interviews and focus groups, to document initial impressions and areas of interest and again during the transcription phase.

As the themes developed, there was a clear divergence of views explained by the degree to which patients were either *satisfied* or *dissatisfied* with their treatment experiences and equally a dichotomy of healthcare practitioners’ experiences that related to *successful* or *unsuccessful* management. This was best expressed by two overarching themes of (1) positive treatment experiences and management (*satisfied* and *successful*) which were founded on education, clear communication and reassurance around prognosis, and (2) negative treatment experiences and management (*dissatisfied* and *unsuccessful*) which was underpinned by uncertainty, delays and exacerbation of pain. Both themes included sub-themes which related to the key areas of; pain, exercise and treatment pathways, but differed in their expression according to their respective overarching theme.

*Theme 1: Positive treatment experience and management*

This theme relates to similar aspects of patient–clinician care that were identified to positively influence the treatment experience and outcome. The overarching theme across all sub-themes was the use of education, clarity in communication and reassurance around expectations and timelines, especially during times of uncertainty. Table 4 provides a summary of the main quotes for *Theme 1* from patients and healthcare practitioners.

**Table 4.** Theme 1: Positive treatment experiences and management.

Sub-themes	Patient quotes	Healthcare practitioner quotes
1. Pain Relief	*‘no, no, just the painkillers. The painkillers were magic’ (Patient 7)*	*‘Pain is always the thing that in my mind you have to control first’ (Surgeon 8)* ‘*The combination of injection plus physio might get them more out of it than they could do just with physio without adequate pain relief’ (Physiotherapist).*
2. Exercise Prescription	*‘doing a bit of painting at home’ (Patient 7)* ‘*swimming in the mornings (Patient 9).* ‘*The physio made all the difference. It absolutely made all the difference. And even to do it right that's the thing and to keep doing it’ (Patient 7).*	*‘hanging out the washing’ (Physiotherapist)* *doing a bit of gardening’* or ‘*just trying to get the plates on the shelf’ (Physiotherapist)* ‘*Your entry point with your exercise on the first session you need to be careful. Starting at the right level. Don’t go too high with these. Just let them get a foot in the door’. (Physiotherapist)* ‘*Keep it simple and give them time’ (Surgeon)*
3. Confidence in their Pathway	*‘I was quite confident with his advice’ (Patient 3)* ‘*I tried everything else but in the end I know surgery was the right thing’ (Patient 8)* ‘*but it's going to be a long road, but I’m happy with this one’ (Patient 11)*	*‘you know that's 6–12 month mark to get that really good function again. I need buy-in for that period of time. So, I’m really clear from the onset’ (Physiotherapist).*

*Sub-theme 1: Pain relief; ‘why they’re happy is they don’t have pain anymore’*

The most important aspect of patient-reported satisfaction with care was achieving good pain control. Patients described how achieving optimal pain relief through the prescription of analgesia was extremely important in helping control their pain levels during the acute presentation or times of an exacerbation. Pain management principally included the use of oral anti-inflammatories/analgesics and/or corticosteroid injections administered by the general practitioner at the first point of contact. The importance of early optimised pain management was echoed by all healthcare practitioners*.* Acute stage pain control aided the participant's ability to move their shoulder and arm and engage successfully with physiotherapy.

*Sub-theme 2: Exercise prescription; ‘Keep it simple and give them time’*

In this theme, patients described the positive impact of exercise therapy as part of either a non-surgical or a surgical pathway and the factors that helped them adhere. Functional, meaningful task execution and goals that aligned with achieving their activities of daily living were essential. Exercise equipment valued by patients included the pulley *‘I think that's a miracle job’ (Patient 1), ‘it's brilliant. I stand with it, I do it every single morning. They are just amazing’ (Patient 3) and Theraband^TM^ ‘was great. It's transportable’ (Patient 8).* When the entry point of exercise selection was optimised and it had become more comfortable to exercise patients felt better when they completed their exercises and could see the difference it made in executing daily tasks. Patients emphasised the importance of doing their exercises, even when their pain and symptoms had settled.

Physiotherapists also echoed the need for exercises to be *a goal more in line with quite simple ADLs’* (Physiotherapist) where the focus was functional with outcomes based on task execution. Shoulder-specific prescription included concepts of support, load, gravity and resistance, and these were all incorporated through mobilisation, close contact positions and flexion plane functional patterns. Physiotherapists emphasised the need to finely balance exercise prescription with other activities of daily living to ensure load management and avoid an exacerbation. Certainly, in a case of less was more, repetition and keeping it simple was the priority. Group-based exercise therapy was highlighted by both surgeons and physiotherapists as an ‘*environment where people could feed off each other and they could encourage each other’.*

*Sub-theme 3: Confidence in their pathway; ‘communication that we are all giving the same message’*

The final sub-theme related to the value placed by patients and healthcare practitioners on a clear treatment pathway and effective patient–practitioner communication. Patients who had a positive experience highlighted that being informed about their management plan including appropriate referral when needed, instilled confidence in them*.* Clarity and explanation as to why one treatment was chosen and not the other were essential to instil adherence, with the confidence in knowing that if the patient ‘*reached a plateau’ (Patient 9)* there were alternative options available. Whilst some patients highlighted their preference against surgery they were also satisfied if they had exhausted other treatment options before agreeing to a surgical intervention*.* Setting early expectations and outcomes enhanced the patient's commitment to the long road ahead and reduced the chance of them questioning and prematurely seeking alternative options.

Healthcare practitioners echoed the importance of clarity from the outset to impart confidence and adherence through setting realistic and achievable timelines, explaining the lengthy period of rehabilitation required. Education, explanation and reassurance around prognosis and what to expect had a very positive effect on patients. For this information, healthcare practitioners concurred that ‘*there is a way to deliver it, and there's a time to deliver it you know? Delivered in a certain way at a certain time is a prognostic indicator’ (General practitioner)*. Consistent communication was highlighted as critical by healthcare practitioners ensuring that it ‘*was the same message’ (Physiotherapist)*, given by all involved and that the language was positive avoiding ‘*non-helpful terminology’ (General practitioner),* for example, ‘*Probably the worst I’ve ever seen’ (Patient 7).*

*Theme 2: Negative treatment experiences and management*

This theme relates to aspects of patient–clinician care that resulted in patient dissatisfaction with elements of care and negatively influenced their experience. While some healthcare practitioners acknowledged factors that had caused poorer outcomes they were not aware of all factors. The overarching theme across all sub-themes was uncertainty, delays and exacerbation of pain. Table 5 provides a summary of the main quotes for *Theme 2* from patients and healthcare practitioners.

**Table 5.** Theme 2: Negative treatment experiences and management.

Sub-themes	Patient quotes	Healthcare practitioner quotes
1. Inappropriate Pain Relief	‘*the pain of it was something else. the morning and through the day. Was hell. Once I was awake’ (Patient 7).* ‘*I went to my GP and he was giving me a painkiller. I said for what I’m not in pain’ (Patient 11)* ‘*my own doctor he was giving me painkillers and that. I don’t take them anymore. There is no point. They don’t do anything. The only thing they do is give me a pain in my stomach you know you’re taking too many’ (Patient 12).*	‘*we stick to what we know, we prescribe lots’ (General Practitioner)*
2. Inadequate Exercise Prescription	*‘but it was when I was doing my exercises it snapped’ (Patient 6)*, *’* *very painful and when I come out then after (physiotherapy) it would be a half an hour, the pain would be in my shoulder, up along here and down there’ (Patient 2)* ‘*but I’d say the type of physio I was doing made it worse’ (Patient 1)*.	‘*increase in pain levels which in their (patient's) head was automatically harm, damage and the whole thing kind of you know not really working.’ (Physiotherapist)*
3. Uncertainty in their Pathway	*‘I don’t think most GPs, they haven’t the experience of what people are suffering with’ (Patient 1)* ‘*That's the first step and that's the block’ (Patient 4).* *They are very negative about it’ (Patient 4)*, ‘*it's so damaged’(Patient 1)*, ‘*probably the worst I’ve ever seen’ (Patient 7*) to ‘*I don’t think he says they can do anything for you’ (Patient 1).*	‘*there's a lot of work to do with GPs about the appropriateness of MRI ordering and interpreting results’ (Surgeon).*

*Sub-theme 1: Inappropriate pain relief; ‘We stick to what we know, we prescribe lots’*

It was acknowledged by patients that early pain management was essential to facilitate progression through care, and, frustration was expressed by patients when their pain was inadequately controlled by their general practitioners which hindered their recovery. However, equally, an over-reliance on the prescription of pain relief as a sole intervention without an appropriate next step was a recurring theme. This over-prescription often resulted in other complications (Gastrointestinal issues) for patients.

An overreliance on the prescription of pain relief was acknowledged by some of the general practitioners ‘*We stick to what we know, we prescribe lots’ (General Practitioner).* Surgeons acknowledged that the next step in care was hindered when the initial acute pain was not well controlled ‘*It's very hard for (physiotherapists) to work on somebody who, you know, pain is not controlled’ (Surgeon)*. This led to an increase in inappropriate surgical referrals due to poor pain management and patients seeking alternative management options ‘*Anything that wasn’t better right away, send off to the consultant’ (Surgeon).*

*Sub-theme 2: Exercise prescription needs to make patients feel better not worse; ‘too much too soon’*

The fine line between getting exercise prescriptions right and wrong was highlighted by patients especially when the entry point of exercise was too advanced resulting in an exacerbation of their pain and negatively impacting their functional ability*.* Patients often suggested that physiotherapy made them worse. It was evident that patients believed they could not engage in an exercise routine that caused them more pain.

‘*I remember now some of the exercises. And it was a man I was with first at the very beginning. And he hurt me. I did say to him, if he thought that I was going to go and hurt myself he had another thing coming’ (Patient 11).*

Healthcare practitioners were quick to point out that often the exercise intervention was either ‘*too tentative an approach or maybe it was too much too soon’ (Physiotherapist).* Some general practitioners acknowledged that they lacked the knowledge and training to get the patient started with some basic exercises and this inexperience may delay the patient's progression. Physiotherapists recognised that common mistakes in exercise prescription were fear around loading or underloading, to the extreme opposite of the spectrum of ‘*being too cavalier about it’* and prescribing too many exercises often not specific enough resulting in symptom exacerbation.

*Sub-theme 3: Uncertainty in their pathway; ‘If we knew, we’d be better’*

Patients who expressed dissatisfaction with their treatment shared a common sub-theme of repeated encounters with healthcare practitioners who lacked sufficient knowledge and confidence in addressing their specific concerns. First-point contact general practitioners were identified as the roadblock to their care due to gaps in their experience and knowledge in managing this cohort. Patients felt their general practitioners should be open to admitting that they didn’t know what to do next which led to delays in their care. Some patients had experiences of physiotherapists not confident enough to treat due to uncertainty ‘*actually afraid, in fairness he said I don’t know what kind of damage is in there until you get this MRI’ (Patient 7)*. Then how information on scan results and prognosis around their care was relayed by some general practitioners negatively impacted patients’ care experience.

Healthcare practitioners could see that patients had lost confidence in their treating practitioners due to the array of mixed messaging and lack of clear expectations. This was detrimental to care and led to delays and sub-optimal care experiences for some patients. Surgeons were frustrated because some general practitioners were requesting scans due to uncertainty, without considering the most appropriate care pathway. Physiotherapists were often viewed as ‘*afraid to do anything because they see the MRI report and they (patients) sit there and do nothing’ (Surgeon)* until they see the surgeon instead of getting them started with an intervention. This approach was described as causing ‘*harm on loads of different levels, delays and at a time when they need to be treated’ (Surgeon)*. Expectations-setting around best care was a ‘*tricky thing with belief system influenced by who they’ve seen before you’ (Physiotherapist).* Surgeons reported that negative messages about tear size and the need for surgery were often communicated by general practitioners and it was difficult ‘*to roll back from that and say the exact opposite’ (Surgeon).*

## Discussion

The findings of this qualitative study have generated new and important information relating to the healthcare experiences of people living with symptomatic large to massive rotator cuff tears, together with the views of clinicians delivering care. It is clear that the positive experiences of care were influenced by effective pain management, being informed of what to expect and the confidence instilled by the treating healthcare practitioners (*theme 1*). This confidence was affirmed by some healthcare practitioners who described the value of their experience and proficiency in their management plan, particularly in times of plateau or poor progress (*theme 1*). Patients’ negative experiences related to reoccurring or persistent pain and a care pathway that lacked clarity and confidence in the treating healthcare practitioners (*theme 2*). Healthcare practitioners acknowledged that a lack of knowledge was associated with this uncertainty. This specifically related to their ability to choose the next step of care and led them to refer for another opinion to deflect this uncertainty (*theme 2*).

The emphasis on the management of pain symptoms in this study echoes the findings of a recent qualitative evidence synthesis of patients’ experiences of a range of shoulder pain conditions where the severe emotional and physical impact of shoulder pain was highlighted,^12^ and the role pain relief had in a recent in-depth thematic analysis exploring decision-making around treatment options for those with rotator cuff tears.^24^ Ten years have passed since Lowe et al. highlighted the need for clinicians to appreciate and understand the intensity and often intolerable nature of pain experienced by their participants (*n* = 20) with symptomatic rotator cuff tears and the detrimental impact these tears have upon all areas of the patient's life. The findings from the current study demonstrate this lack of understanding remains the experience for the majority of this patient cohort.

Exercise is often prescribed in the non-operative, pre-operative and post-operative treatment of large to massive rotator cuff tears. A recent systematic review with a synthesis of a standardised protocol concluded that despite low-quality evidence and vast variations in the type of exercise, good clinical outcomes could be achieved in non-operative management.^7^ This echoed the positive responses from some of the non-operative participants in the current study but also our surgical cohort on their experience of their functional achievements following an exercise intervention both pre- and post-surgery. Exacerbation of pain was the main reason participants reported negative experiences during the rehabilitation process. It has previously been reported in the literature^24^ for those with rotator cuff tears of all sizes that exercise (type not specified) often provoked their symptoms (*n* = 15), causing pain and fear of further damage which led to a decision to not exercise or undergo surgery often prematurely. The findings from the current study indicate that several factors may contribute to the patient's positive or negative experience of exercise for their condition, that is, expertise of the clinician, exercise entry point and level and education around managing the overall load.

The sub-optimal management of pain and lack of guidance around exercise parameters highlight the prevalence of uncertainty about how best to manage these tears. This leads to the patient being exposed to mixed messaging causing annoyance, frustration and a loss of confidence in their respective treatment plan. Recent research on the importance of an informed healthcare relationship^24^ concluded that almost half of the 15 people diagnosed with a rotator cuff tear in the study were satisfied that the information they received was adequate to help inform their decision around their healthcare*.* Providing adequate information to people with rotator cuff tears may improve patient-centred care, especially about premature decision-making around surgery. An inductive qualitative study with eight participants with rotator cuff-related shoulder pain highlighted the importance of a therapeutic alliance to build trust around the information provided. Education needs to be individualised and practical for the patient, and the mode of delivery of educational interventions should be varied and multi-modal.^25^ Our patients reported positive experiences where they trusted the healthcare practitioners providing the education, which facilitated adherence and increased belief that their condition was being effectively treated.

The varying opinions expressed by healthcare practitioners in our study were based on their level of confidence, knowledge and experience with large to massive rotator cuff tears. In a study exploring expert shoulder clinicians’ (*n* = 8) management of rotator cuff tendinopathy, it was identified that early focused education was a fundamental component of management and plays a vital role in developing a strong patient–therapist relationship and instilling trust in the healthcare practitioners’ management plan.^26^ The lack of clear guidance on the best management of this condition leads to this lack of confidence in clinicians.^10^ Through insights gained from this qualitative research, healthcare practitioners can be supported to recognise gaps in care and develop solutions to improve outcomes.

The strengths of this study were the purposive sample of people treated for large to massive rotator cuff tears and their treating healthcare practitioners interviewed for this qualitative study. Included clinicians who had a range of experience across three different disciplines and were practising in varying proportions in private and public healthcare in Ireland. The interviewer endeavoured to be reflexive about their influence on data collection, to mitigate against any potential bias relating to the clinical background. Measures taken to mitigate this include an interview guide based on current research to uniform information gathering, appropriate qualitative training and regular debriefing between the interviewers and the research team. In terms of limitations, these findings must be interpreted in light of the self-selected sample of participants who volunteered for this study as patients of the included healthcare practitioners knowing that the interviewer was a practising physiotherapist. It is possible they responded in a socially desirable manner by over-emphasising the role of exercise and physiotherapy in their care pathway. Our findings may be affected by recall bias (recalling the treatment and education they received) given that patient focus groups were conducted up to one year post-surgery.

The study explored the treatment experiences of those diagnosed with symptomatic large to massive rotator cuff tears and the perspectives of healthcare practitioners providing this care. We found a disconnect between the needs and expectations of patients with large to massive rotator cuff tears and the care they received from healthcare practitioners, particularly in the areas of pain management, education and exercise prescription. HCPs acknowledged a lack of education to instil confidence in their management strategies. This highlights a need for improved education, clinical practice guidelines and standardising care for patients with large to massive rotator cuff tears.

**Clinical messages**

- Good first-contact pain management is vital for the progression of the patient care pathway.
- An intervention grounded in education instilled confidence.
- Healthcare practitioners identified the gap in knowledge and resultant confidence in treating large to massive rotator cuff tears.

## Supplemental Material

sj-docx-1-cre-10.1177_02692155241235338 - Supplemental material for ‘If he thought that I was going to go and hurt myself, he had another thing coming’: Treatment experiences of those with large to massive rotator cuff tears and the perspectives of healthcare practitioners Supplemental material, sj-docx-1-cre-10.1177_02692155241235338 for ‘If he thought that I was going to go and hurt myself, he had another thing coming’: Treatment experiences of those with large to massive rotator cuff tears and the perspectives of healthcare practitioners by Kathryn Fahy, Rose Galvin, Jeremy Lewis and Karen McCreesh in Clinical Rehabilitation

sj-docx-2-cre-10.1177_02692155241235338 - Supplemental material for ‘If he thought that I was going to go and hurt myself, he had another thing coming’: Treatment experiences of those with large to massive rotator cuff tears and the perspectives of healthcare practitioners Supplemental material, sj-docx-2-cre-10.1177_02692155241235338 for ‘If he thought that I was going to go and hurt myself, he had another thing coming’: Treatment experiences of those with large to massive rotator cuff tears and the perspectives of healthcare practitioners by Kathryn Fahy, Rose Galvin, Jeremy Lewis and Karen McCreesh in Clinical Rehabilitation

## References

[1] Teunis T Lubberts B Reilly BT , et al. A systematic review and pooled analysis of the prevalence of rotator cuff disease with increasing age. J Shoulder Elbow Surg 2014; 23: 1913–1921. 25441568 10.1016/j.jse.2014.08.001

[2] Tempelhof S Rupp S Seil R . Age-related prevalence of rotator cuff tears in asymptomatic shoulders. J Shoulder Elbow Surg 1999; 8: 296–299. 10471998 10.1016/s1058-2746(99)90148-9

[3] Shim SB Jeong JY Kim JS , et al. Evaluation of risk factors for irreparable rotator cuff tear in patients older than age 70 including evaluation of radiologic factors of the shoulder. J Shoulder Elbow Surg 2018; 27: 1932–1938. 30340802 10.1016/j.jse.2018.07.011

[4] Edwards P Ebert J Joss B , et al. Exercise rehabilitation in the non-operative management of rotator cuff tears: a review of the literature. Int J Sports Phys Ther 2016; 11: 279. 27104061 PMC4827371

[5] Minns Lowe CJ Moser J Barker K . Living with a symptomatic rotator cuff tear ‘bad days, bad nights’: a qualitative study. BMC Musculoskelet Disord 2014; 15: –0. 10.1186/1471-2474-15-228 PMC4105791 25008095

[6] Littlewood C May S Walters S . A review of systematic reviews of the effectiveness of conservative interventions for rotator cuff tendinopathy. Shoulder Elbow 2013; 5: 151–167.

[7] Shepet KH Liechti DJ Kuhn JE . Nonoperative treatment of chronic, massive irreparable rotator cuff tears: a systematic review with synthesis of a standardized rehabilitation protocol. J Shoulder Elbow Surg 2021; 30: 1431–1444. 33276163 10.1016/j.jse.2020.11.002

[8] Gerber C Wirth SH Farshad M . Treatment options for massive rotator cuff tears. J Shoulder Elbow Surg 2011; 20: S20–S29. 10.1016/j.jse.2010.11.028 21281919

[9] Kovacevic D Suriani RJ Jr Grawe BM , et al. Management of irreparable massive rotator cuff tears: a systematic review and meta-analysis of patient-reported outcomes, reoperation rates, and treatment response. J Shoulder Elbow Surg 2020; 29: 2459–2475. 32763381 10.1016/j.jse.2020.07.030 PMC7669555

[10] Fahy K Galvin R Lewis J , et al. Exercise as effective as surgery in improving quality of life, disability, and pain for large to massive rotator cuff tears: a systematic review & meta-analysis. Musculoskeletal Sci Pract 2022; 61: 102597. DOI: 10.1016/j.msksp.2022.102597. 35724568

[11] Beaton DE Clark JP . Qualitative research: a review of methods with the use of examples from the total knee replacement literature. JBJS 2009; 91: 107–112. 10.2106/JBJS.H.01631 19411508

[12] Maxwell C Robinson K McCreesh K . Understanding shoulder pain: a qualitative evidence synthesis exploring the patient experience. Phys Ther 2021; 101: pzaa229. 10.1093/ptj/pzaa229 33373455

[13] Braun V Clarke V . One size fits all? What counts as quality practice in (reflexive) thematic analysis? Qual Res Psychol 2021; 18: 328–352.

[14] Malterud K Siersma VD Guassora AD . Sample size in qualitative interview studies: guided by information power. Qual Health Res 2016; 26: 1753–1760. 26613970 10.1177/1049732315617444

[15] Tong A Sainsbury P Craig J . Consolidated Criteria for Reporting Qualitative Research (COREQ): a 32-item checklist for interviews and focus groups. Int J Qual Health Care 2007; 19: 349–357. 17872937 10.1093/intqhc/mzm042

[16] Suri H . Purposeful sampling in qualitative research synthesis. Qual Res J 2011; 11: 63–75.

[17] Cohen N Arieli T . Field research in conflict environments: methodological challenges and snowball sampling. J Peace Res 2011; 48: 423–435.

[18] Leighton K Kardong-Edgren S Schneidereith T , et al. Using social media and snowball sampling as an alternative recruitment strategy for research. Clin Simul Nurs 2021; 55: 37–42.

[19] French HP Galvin R . Musculoskeletal services in primary care in the republic of Ireland: an insight into the perspective of physiotherapists. Physiotherapy 2017; 103: 214–221. 27650298 10.1016/j.physio.2016.05.007

[20] King WV Hebron C . Frozen shoulder: living with uncertainty and being in “no-man’s land”. Physiother Theory Pract 2022; 39: 1–5. 10.1080/09593985.2022.2032512 35164645

[21] Braun V Clarke V . Reflecting on reflexive thematic analysis. Qual Res Sport Exerc Health 2019; 11: 589–597.

[22] Ritchie J Lewis J Nicholls CM Ormston R . Qualitative research practice: A guide for social science students and researchers. London: Sage; 2013.

[23] Byrne D . A worked example of Braun and Clarke’s approach to reflexive thematic analysis. Qual Quant 2022; 56: 1391–1412.

[24] Malliaras P Rathi S Burstein F , et al. Physio’s not going to repair a torn tendon’: patient decision-making related to surgery for rotator cuff related shoulder pain. Disabil Rehabil 2022; 44: 3686–3693. 33577359 10.1080/09638288.2021.1879945

[25] Cridland K Pritchard S Rathi S , et al. He explains it in a way that I have confidence he knows what he is doing’: a qualitative study of patients’ experiences and perspectives of rotator-cuff-related shoulder pain education. Musculoskeletal Care 2021; 19: 217–231. 33258225 10.1002/msc.1528

[26] White J Mc Auliffe S Jepson M , et al. There is a very distinct need for education among people with rotator cuff tendinopathy: an exploration of health professionals’ attitudes. Musculoskeletal Sci Pract 2020; 45: 102103. 10.1016/j.msksp.2019.102103 32056827

