# Supplementary material for: ‘If he thought that I was going to go and hurt myself, he had another thing coming’: Treatment experiences of those with large to massive rotator cuff tears and the perspectives of healthcare practitioners
Source: Clin Rehabil. 2024 Feb 28;38(6):824–36. doi: 10.1177/02692155241235338 (PMC11059833; doi:10.1177/02692155241235338)
Supplement: sj-docx-1-cre-10.1177_02692155241235338 - Supplemental material for ‘If he thought that I was going to go and hurt myself, he had another thing coming’: Treatment experiences of those with large to massive rotator cuff tears and the perspectives of healthcare practitioners [file sj-docx-1-cre-10.1177_02692155241235338.docx]

**Interview Guide: Individuals with L-MRCTTs**

Today we want to discuss **your ‘lived experience’** in managing your shoulder pain and your treatment experience to date

- Tell me what it’s like to live with shoulder pain?
- Can you elaborate on..
- What do you believe is causing the pain or the problem?
- Besides your shoulder what other factors do you think influence your pain daily?

Next I want to look at **your treatmet experience to date**

- What have been the positive aspects of your treatment?
- What aspects of your treatment would you change?
- What was your role in deciding your treatment to date?

Now I want to discuss the **practical aspects** of your exercise intervention

- Did you undertake an exercise programme
- What worked well?
- What didn’t work well?

Finally, I want to **discuss,**

- What you would change to improve your experience of treatment
- What would you change to help try improve your pain, function and Quality of Life
- If you had your treating practitioner present would you say or want to do anything differently.

To conclude, is there anything else you would like to add to the discussion about your shoulder pain and your healthcare experience to date?

**Interview guide: HCPs (Orthopedic surgeons, Physiotherapists and GP’s)**

Today we want to discuss your **experiences in managing** people with Large to massive rotator cuff tedon tears.

- What is indicative of good outcomes? Poorer outcomes?
- What is your rational for its influence?

Next I want to discuss your **criteria for choosing your treatment pathway**

- What are your beliefs around best management or key interventions for this cohort?
- Do you follow certain criteria (S) guidelines/outcomes (PT) to help inform your treatment decision, if so what were they?
- How much of a role did your patient play in the treatment choice? Influence of the Irish Health System (Public/Private system)

.

- What do you believe is the role of exercise in managing this problem?

**Finally,**

- What have you learned from managing these patients? Has it changed how you plan your patient care?
- What can we do better?

To conclude, is there anything else you would like to add to the discussion on managing people with L-MRCCTs?
