# Supplementary material for: ‘If he thought that I was going to go and hurt myself, he had another thing coming’: Treatment experiences of those with large to massive rotator cuff tears and the perspectives of healthcare practitioners
Source: Clin Rehabil. 2024 Feb 28;38(6):824–36. doi: 10.1177/02692155241235338 (PMC11059833; doi:10.1177/02692155241235338)
Supplement: sj-docx-2-cre-10.1177_02692155241235338 - Supplemental material for ‘If he thought that I was going to go and hurt myself, he had another thing coming’: Treatment experiences of those with large to massive rotator cuff tears and the perspectives of healthcare practitioners [file sj-docx-2-cre-10.1177_02692155241235338.docx]

Consolidated Criteria for Reporting Qualitative Research (COREQ) 32- item checklist (Tong et al 2007)

| **No. Item** | **Guide questions/description** | **Reported on Page #** |
| --- | --- | --- |
| **Domain 1: Research team and reﬂexivity** |  |  |
| *Personal Characteristics* |  |  |
| 1. Inter viewer/facilitator | Which author/s conducted the interview or focus group? | Page 8/ paragraph 2 |
| 2. Credentials | What were the researcher’s credentials? E.g., PhD, MD | Page 8/ paragraph 2 (Line) |
| 3. Occupation | What was their occupation at the time of the study? | Page 8 / paragraph 2 (Line) |
| 4. Gender | Was the researcher male or female? | Page 8 / paragraph 2  (Line) |
| 5. Experience and training | What experience or training did the researcher have? | Page 8 / paragraph 2  (Line) |
| *Relationship with participants* |  |  |
| 6. Relationship established | Was a relationship established prior to study commencement? | Page 8 / paragraph 2  **No** |
| 7. Participant knowledge of the interviewer | What did the participants know about the researcher? e.g., personal goals, reasons for doing the research | Page 8 / paragraph 2  (Line) |
| 8. Interviewer characteristics | What characteristics were reported about the inter viewer/facilitator? e.g., Bias, assumptions, reasons, and interests in the research topic | Page 8 / paragraph 2  (Line) |

| **Domain 2: study design** |  |  |
| --- | --- | --- |
| *Theoretical framework* |  |  |
| 9. Methodological orientation and Theory | What methodological orientation was stated to underpin the study? e.g., grounded theory, discourse analysis, ethnography, phenomenology, content analysis | Page 5 & 8 / paragraph 3  (Line) |
| *Participant selection* |  |  |
| 10. Sampling | How were participants selected? e.g., purposive, convenience, consecutive, snowball | Page 5 / paragraph 4  (Line) |
| 11. Method of approach | How were participants approached? e.g., face-to-face, telephone, mail, email | Page 7 / paragraph 2  (Line) |
| 12. Sample size | How many participants were in the study? | Page 6 / paragraph 1 & 3 (Line) |
| 13. Non-participation | How many people refused to participate or dropped out? Reasons? | **None** |
| *Setting* |  |  |
| 14. Setting of data collection | Where was the data collected? e.g., home, clinic, workplace | Clinic, hospital meeting room and online |
| 15. Presence of non-participants | Was anyone else present besides the participants and researchers? | **No** |
| 16. Description of sample | What are the important characteristics of the sample? e.g., demographic data, date | Page 17 Table 1 & 2 |
| *Data collection* |  |  |
| 17. Interview guide | Were questions, prompts, guides provided by the authors? Was it pilot tested? | Page 7/8 / paragraph 3 (Line) |
| 18. Repeat interviews | Were repeat inter views carried out? If yes, how many? | **No** |
| 19. Audio/visual recording | Did the research use audio or visual recording to collect the data? | Page 7 / paragraph 2 (Line) |
| 20. Field notes | Were ﬁeld notes made during and/or after the interview or focus group? | Page 8 / paragraph 2 |
| 21. Duration | What was the duration of the inter views or focus group? | Page 8 / paragraph 2(Line) |
| 22. Data saturation | Was data saturation discussed? | Page 5 / paragraph 3(Line) |
| 23. Transcripts returned | Were transcripts returned to participants for comment and/or correction? | **No** |
|  |  |  |
| **Domain 3: analysis and ﬁndings** |  |  |
| *Data analysis* |  |  |
| 24. Number of data coders | How many data coders coded the data? | **Three** |
| 25. Description of the coding tree | Did authors provide a description of the coding tree? | Page 9 / paragraph 2 (Line) |
| 26. Derivation of themes | Were themes identiﬁed in advance or derived from the data? | Page 5 / paragraph 3(Line)  **Themes were derived from the data** |
| 27. Software | What software, if applicable, was used to manage the data? | **NVivo** (Version 12) |
| 28. Participant checking | Did participants provide feedback on the ﬁndings? | **No** |
| *Reporting* |  |  |
| 29. Quotations presented | Were participant quotations presented to illustrate the themes/ﬁndings? Was each quotation identiﬁed? e.g., participant number | Yes |
| 30. Data and ﬁndings consistent | Was there consistency between the data presented and the ﬁndings? | Page / paragraph (Line) ~ Page / paragraph (Line)  **Yes** |
| 31. Clarity of major themes | Were major themes clearly presented in the ﬁndings? | Page / paragraph (Line) ~ Page / paragraph (Line)  **Yes** |
| 32. Clarity of minor themes | Is there a description of diverse cases or discussion of minor themes? | Page / paragraph (Line) ~ Page / paragraph (Line)  **Yes** |
